# Palladium(II) Complexes Containing Mixed Nitrogen-Sulphur Donor Ligands: Interaction of [Pd(Methionine Methyl Ester)(H_2_O)_2_]^2+^ with Biorelevant Ligands

**DOI:** 10.1155/2014/382646

**Published:** 2014-08-24

**Authors:** Mohamed M. Shoukry, Sameya M. T. Ezzat

**Affiliations:** ^1^Department of Chemistry, Faculty of Science, Islamic University, Madinah, Saudi Arabia; ^2^Department of Chemistry, Faculty of Science, Cairo University, P.O. Box 12613, Giza, Egypt

## Abstract

Pd(MME)Cl_2_ complex (MME = methionine methyl ester) was synthesised and characterized by physicochemical measurements. The reaction of [Pd(MME)(H_2_O)_2_]^2+^ with amino acids, peptides, or dicarboxylic acids was investigated at 25°C and 0.1 M ionic strength. Amino acids and dicarboxylic acids form 1 : 1 complexes. Peptides form both 1 : 1 complexes and the corresponding deprotonated amide species. The stability of the complexes formed was determined and the binding centres of the ligands were assigned. Effect of solvent on the stability constant of Pd(MME)-CBDCA complex, taken as a representative example, shows that the complex is more favoured in a medium of low dielectric constant. The concentration distribution diagrams of the complexes were evaluated.

## 1. Introduction

cis-platin [cis-diamminedichloroplatinum(II)] is one of the most active antitumor agents in clinical use [1]. However, it has a narrow spectrum of activity, and its clinical use is limited by undesirable side effects, including nephrotoxicity, ototoxicity, nausea, vomiting, and myelosuppression. Significant efforts were made to produce novel platinum-containing compounds in order to improve structural and physiological disadvantages, such as several side effects, drug resistance, and limitation in the field of application. Most of the developed and studied platinum complexes include nitrogen-containing monodentate, bidentate, or tridentate ligands [2–8]. The ultimate aim of the modifications of the parent drug is to make related analogues that produce a different spectrum of DNA lesions and so circumvent the problem of resistance to cisplatin [2, 9] Therefore, nonclassical platinum derivatives were also investigated, which may violate the classical structure-activity relationship [2]. Complexes include sulphur-containing ligands and show antitumor activity [10]. An example is [Pt(CH_3_SCH_2_CH_2_SCH_3_)Cl_2_], Pt(dt), a sulphur analogue of the well-studied [Pt(H_2_NCH_2_CH_2_NH_2_)Cl_2_], Pt(en)Cl_2_ complex [10]. Baltic et al. examined the antitumor activity of Pt(dt) against the human breast cancer cell line and found the complex to inhibit the growth of MCF-7 cells in a dose and time dependent manner. Another example is the complex dichloro(2-methylthiomethylpyridine)platinum(II)Pt(mtp)Cl_2_ a complex with nitrogen as well as sulphur donor ligands. In the literature different synthetic pathways are described [11–13] and the bidentate N, S-complex was found to be promising cytostatic agent [14].

Pd(II) and Pt(II) amine complexes have the same structure, with five orders of magnitude higher reactivity in the case of Pd(II) complexes, but similar thermodynamic parameters. Pd(II) complexes are good models for the analogous Pt(II) complexes in solution. Recent work in our laboratories focused on the kinetics and equilibria of complex-formation reactions of cis-(diamine)palladium(II) complexes with DNA, the major target in chemotherapy of tumours, and biorelevant ligands as amino acids, peptides, dicarboxylic acids, and esters [15–18]. The present investigation describes the equilibria associated with the interaction of [Pd(MME)(H_2_O)_2_]^2+^ with biorelevant ligands as amino acids, peptides, and dicarboxylic acids. The results may give information regarding the distribution of the antitumor agent in biological fluid.

## 2. Experimental

### 2.1. Materials and Reagents

All reagents were of analytical grade. K_2_PdCl_4_ and Methionine methyl ester hydrochloride were obtained from Aldrich. The amino acids and related compounds (glycine, alanine, *β*-phenylalanine, proline, methionine imidazole, lysine, histidine, and histamine) were provided by Sigma Chemical Co. The peptides and amides used (glycinamide, glycylglycine, asparagine, and glutamine) and the dibasic acids used (cyclobutane 1,1-dicarboxylic acid, oxalic acid, malonic acid, succinic acid, and adipic acid) were all provided by BDH-Biochemicals Ltd., Poole, England. For equilibrium studies, [Pd(MME)Cl_2_] was converted into the diaqua complex by treating it with two equivalents of AgNO_3_ as described before [15]. The ligands in the form of hydrochlorides were converted into the corresponding hydronitrates [15] All solutions were prepared in deionized water.

### 2.2. Synthesis

Pd(MME)Cl_2_ was prepared by dissolving K_2_PdCl_4_ (2 mmol) in 10 mL water with stirring. The clear solution of [PdCl_4_]^2-^ was filtered and Methionine methyl ester hydrochloride (2 mmol), dissolved in 10 mL H_2_O, was added dropwise to the stirred solution. A yellowish-brown precipitate of [Pd(MME)Cl_2_] was formed and stirred for a further 30 minutes at 50°C. After filtering of the precipitate. A yellow powder was obtained. An orange crystalline precipitate was obtained; yield 98.9%. Anal. Calcd., for PdC_6_H_13_SNO_2_Cl_2_ (*F*. Wt = 340.38): C, 21.16; H, 3.81; N, 4.11; S, 9.40. Found: C, 21.2; H, 4.04; N, 3.7; S, 9.1.

### 2.3. Apparatus

Potentiometric titrations were performed with a Metrohm 686 titroprocessor equipped with a 665 Dosimat. The titroprocessor and electrode were calibrated with standard buffer solutions, prepared according to NBS specification [19]. The pH meter readings were converted to hydrogen ion concentration by titrating a standard HNO_3_ solution (0.01 M), the ionic strength of which was adjusted to 0.1 M with NaNO_3_, with standard NaOH (0.05 M). The pH was plotted against p[H]. The relationship pH − p[H] = 0.05 was observed. All titrations were carried out at 25.0 ± 0.1°C in purified nitrogen atmosphere using a titration vessel described previously [20]. The p*K*_w_ in dioxane-water solution was determined as described previously [16, 17]. For this purpose, various amounts of standard NaOH solution were added to a solution containing 0.1 M NaNO_3_. The [OH^−^] was calculated from the amount of base added. The [H^+^] was calculated from the pH value. The product of [OH^−^] and [H^+^] was taken. IR spectra were measured on an 8001-PC FT-IR Shimadzu spectrophotometer using KBr pellets.

### 2.4. Procedure and Measuring Technique

The acid dissociation constants of the ligands were determined by titrating 1 mmole samples of each with standard NaOH solutions. Ligands were converted into their protonated form with standard HNO_3_ solutions. The acid dissociation constants of the coordinated water molecules in [Pd(MME)(H_2_O)_2_]^2+^ were determined by titrating 1 mmole of complex with standard 0.05 M NaOH solution. The formation constants of the complexes were determined by titrating solution mixtures of [Pd(MME)(H_2_O)_2_]^2+^ (1 mmole) and the ligand in the concentration ratio of 1 : 1 for amino acids, peptides, and dicarboxylic acids. The titrated solution mixtures each had a volume of 40 mL and the titrations were carried out at 25°C and 0.1 M ionic strength (adjusted with NaNO_3_), such a condition is similar to what exists in biological system. A standard 0.05 M NaOH solution was used as titrant.

The species formed were characterized by the general equilibrium

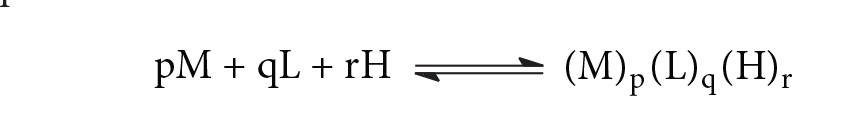
(1)
for which the formation constants are given by

(2) $$\begin{array}{c} \beta_{\text{pqr}}=\frac{\left[{\left(\text{M}\right)}_{\text{p}}{\left(\text{L}\right)}_{\text{q}}{\left(\text{H}\right)}_{\text{r}}\right]}{{\left[\text{M}\right]}^{\text{p}}{\left[\text{L}\right]}^{\text{q}}{\left[\text{H}\right]}^{\text{r}}} \end{array}$$

where M, L, and H stand for [Pd(MME)(H_2_O)_2_]^2+^ ion, ligand, and proton, respectively. The calculations were performed using the computer program [21] MINIQUAD-75. The stoichiometry and stability constants of the complexes formed were determined by trying various possible composition models for the systems studied. The model selected was that which gave the best statistical fit and was chemically consistent with the magnitudes of various residuals, as described elsewhere [21]. The accepted model gave smallest standard deviation and sum of square of residuals. The reliability of the proposed model is tested by comparing the experimental titration data and the theoretically simulated data obtained by MINIQUAD-75 program.

Tables 1, 2, 3, and 4 list the stability constants together with their standard deviations and the sum of the squares of the residuals derived from the MINIQUAD output. The concentration distribution diagrams were obtained with the program SPECIES [22] under the experimental condition used.

## 3. Results and Discussion

The analytical data indicated that the complex is of 1 : 1 stoichiometry and of formula Pd(MMA)Cl_2_. From comparing the IR spectrum of MME and Pd(MME)Cl_2_ complex, the stretching vibration of NH group of methionine methyl ester located at 2954 cm^−1^ is broadened in the complex spectrum due to coordination by the amino group. The ester carbonyl group of the ligand exhibits a sharp band at 1745 cm^−1^. This band was not affected upon complex formation. This indicates that the ester group is not participated in complex formation and methionine methyl ester is bound to Pd(II) by the amino and sulphur atom.

Before investigating the complex formation equilibria involving [Pd(MME)(H_2_O)_2_]^2+^ the hydrolysis of the ester group of methionine methyl ester was tested by pH-stat technique [17]. The results show that the extent of ester hydrolysis in the pH range of the complex formation study is very small and can be neglected.

### 3.1. Acid-Base Equilibria of [Pd(MME)(H_2_O)_2_]^2+^

The acid-base equilibria of the [Pd(MME)(H_2_O)_2_]^2+^ complex is characterized by fitting the potentiometric data to various models. The best fit model was found to be consistent with species 10-1, 10-2, and 20-1. The first two species, 10-1 and 10-2, are due to deprotonation of the two coordinated water molecules as given by (3) and (4).

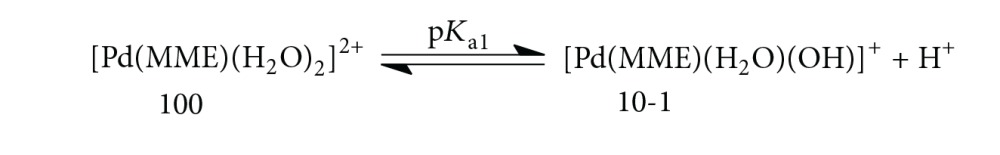
(3)

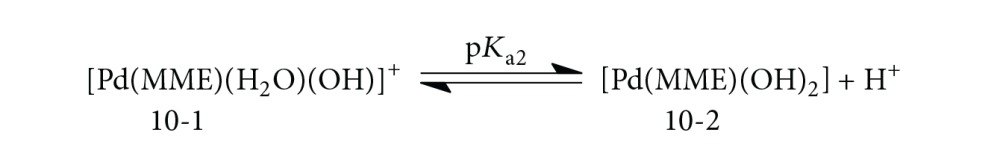
(4)
The third species, 20-1, is the dimeric species formed as a result of the combination of the monoaqua hydroxo species (10-1) with the diaqua species (100) [Pd(MME)(H_2_O)_2_]^2+^ as given by:

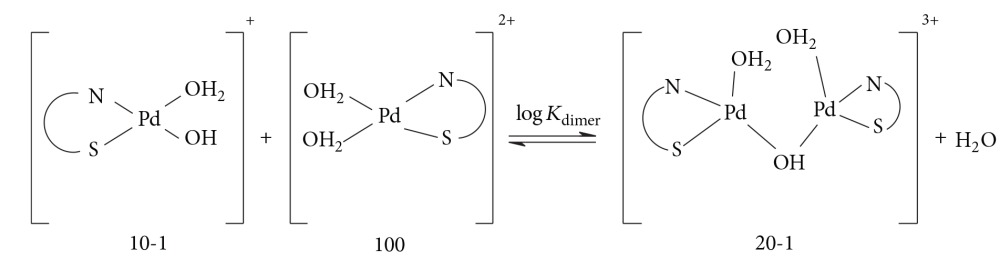
(5)


p*K*_a1_ and p*K*_a2_ values for [Pd(MME)(H_2_O)_2_]^2+^ were found to be 5.29 and 7.67, respectively. The equilibrium constant value of the dimerisation reaction, given in (5) and calculated by (6), is 2.83:

(6) $$\begin{array}{c} {\text{Log}K}_{\text{dimer}}=\log\beta_{20\text{-}1}-\log\beta_{10\text{-}1} \end{array}$$

The formation of the dihydroxo-bridged dimer (20-2), found for most Pd-diimine complexes, is not favoured in the case of the Pd-MME complex. This may be accounted for on the basis that the strong labilization effect of the S-donor atom will cause the dimeric form (20-2) to be strained and consequently energetically not favoured [17].

**Figure 1:**
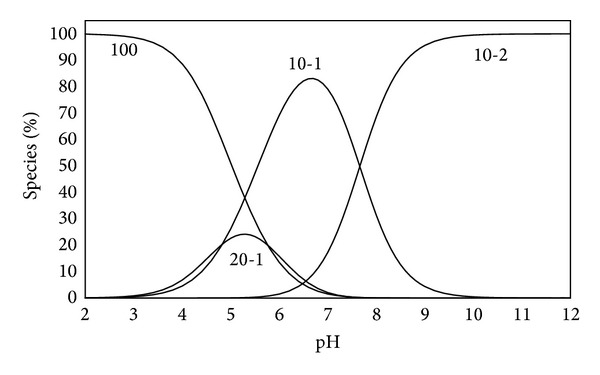
Concentration distribution of various species as a function of pH in the Pd(MME)-OH system (at concentration of 1.25 mmole/liter for Pd(MME)).

The concentration distribution diagram for [Pd(MME)(H_2_O)_2_]^2+^ and its hydrolysed species is shown in Figure 1. The concentration of monohydroxo species, 10-1, and the dimeric species increase with increasing pH. The dimeric species, 20-1, attains maximum concentration of ~24% at pH ~5.2. The monohydroxo species (10-1) is the main species in the pH rang ~5.5 to 7.5; that is, it is the main species present in the physiological conditions. A further increase in pH is accompanied by an increase in the dihydroxo species (10-2) concentration, which is the main species above pH ca.8.

### 3.2. Complex Formation Equilibria Involving Amino Acids

Fitting of pH titration data for Pd(MME)-amino acid equilibria indicated the formation of 1 : 1 complexes with high stability constant values. This indicates that amino acids bind through the amino and carboxylate groups. Histidine is a tridentate ligand having amino, imidazole, and carboxylate groups as binding sites. With [Pd(MMA)(H_2_O)_2_]^2+^, only two of the three binding sites are involved in the complex formation; hence histidine coordinates in either a glycine-like or a histamine-like mode. The stability constant of the histidine complexes is significantly higher than that of histamine and amino acids. This indicates that histidine interacts with the Pd^II^ complex by the amino and imidazole groups. The proline complex has the highest value among the simple amino acids. This may be due to the highest basicity of the proline amino group as reflected by the highest p*K*_a_ value. The stability constant of lysine complex is not so higher, as compared with the stability constants of the other amino acids. This may be explained on the premise that lysine coordinates by the two amino groups forming a less stable eight-membered chelate ring. The stability of methionine complex (log*β* = 9.05) is lower than that of most simple amino acids. This may be explained on the premise that the amino group of methionine is less basic than that of other amino acids as reflected by p*K*_a_ values.

### 3.3. Complex Formation Equilibria Involving Peptides

The potentiometric data of the peptide (HL) complexes were fitted assuming the formation of the species [Pd(MME)(L)]^+^ (110 species) and [Pd(MME)(LH_−1_)] (11-1 species). The former species is formed by coordination through the amino group and carbonyl oxygen atom. On increasing the pH, the coordination site should switch from the carbonyl oxygen to the amide nitrogen with release of the amide hydrogen, forming the complex [Pd(MME)(LH_−1_)]. Such changes in coordination centers are now well documented [23, 24]. The p*K* ^H^ of the coordinated amide group was calculated using (7) and is given in Table 2:

(7) $$\begin{array}{c} {\text{p}K}^{\text{H}}=\log\beta_{110}-\log\beta_{11\text{-}1} \end{array}$$

The p*K* ^H^ for the glycinamide complex is lower than that of the other peptides. This may be explained on the premise that the more bulky substituent group on the peptide may serve to hinder the structural change in going from protonated to deprotonated complexes. Asparagine complex has the highest stability constant value, most probably due to the presence of *α*-amino group that can coordinate firstly as glycine does. The *α*-amino group of asparagine is more basic than that of other peptides, which result in an increase of stability constant of its complex.

The concentration distribution diagrams of peptide complexes indicate that all peptides form the complex species (110) at low pH and thus prevent the hydrolysis of Pd(II) ion; that is, the hydrolysed species (10-1) and (10-2) are either not formed or formed in very low concentration. The induced ionization of the peptide hydrogen of glycinamide and glycylglycine starts at pH ~3. However asparagine ionization starts above pH ~8. Therefore, under normal physiological condition (pH 6-7), the peptides would coordinate to [Pd(MME)(H_2_O)_2_]^2+^ in entirely different ways. Glycinamide and glycylglycine are present entirely in the deprotonated form (11-1), whereas asparagine exists solely in the protonated form.

### 3.4. Complex Formation Equilibria Involving Dicarboxylic Acids

**Figure 2:**
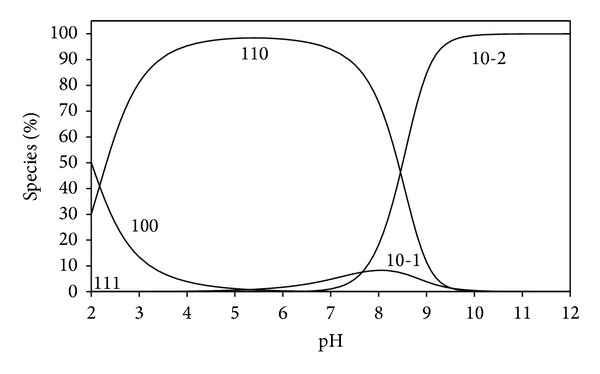
Concentration distribution of various species as a function of pH in the Pd(MME)-CBDCA system (at concentration of 1.25 mmole/liter for Pd(MME) and CBDCA).

In the case of dicarboxylic acids, the potentiometric data were fitted on the basis of the formation of 110 complex and its monoprotonated form (111). The formation constants of the (110) complexes formed with oxalic, CBDCA and malonic acids, where five- and six-membered chelate rings are higher than those involving seven membered, as in succinic, and nine-membered chelate rings as in adipic acid. This may be explained on the premise that the five- and six-membered rings are more favored energetically than the seven- and the nine-membered rings. It is interesting to note that 1,1-CBDCA has a higher stability constant than that of malonic acid, although both form 6-membered chelate rings. This may be due to the higher p*K*_a_ values of the former than the latter dicarboxylic acid. The p*K*_a_ value of the protonated complex species of [Pd(MME)(CBDCA)] is 1.82. This value is lower than that of free CBDCAH-, which indicates acidification of the second carboxylic group upon coordination of [Pd(MME)(H_2_O)_2_]^2+^ with the first carboxylate group. The p*K*_a_ value of this protonated species in case of [Pd(en)HCBDCA]^+^ was estimated before from u.v./vis measurements to be ca.2.5 at 25°C and 0.1 M ionic strength [25]. This species was documented to be the active form in the case of carboplatin [26]. The concentration distribution diagram of CBDCA complex, taken as an example, is given in Figure 2 and shows that the protonated species (111) is stable only at low pH and the species (110) is predominating at the physiological pH. The hydrolysed species for all dibasic acid complexes predominates only at high pH.

### 3.5. Effect of Solvent on Complex Formation Equilibria

It was reported that a lower polarity has been detected in some biochemical microenvironments, such as active sites of enzymes and side chains in proteins [27–30]. These properties approximately correspond to those (or can be simulated by those) existing in the water/dioxane mixtures. Consequently, a study of the Pd(MME)-CBDCA complex formation, taken as a typical example, in dioxane-water solutions of different compositions, could be of biological significance. In order to characterize the formation equilibria of the Pd(MME)-CBDCA complex in dioxane-water solutions, all other equilibria involved, namely, acid-base equilibria of CBDCA and [Pd(MME)(H_2_O)_2_]^2+^, have to be studied in the same solvent. The equilibrium constants are reported in Table 4.

**Figure 3:**
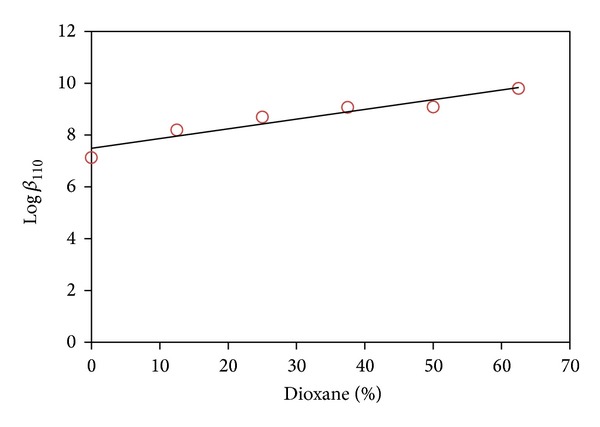
Effect of dioxane on the CBDCA complex formation constant, Log*β*_110_.

**Figure 4:**
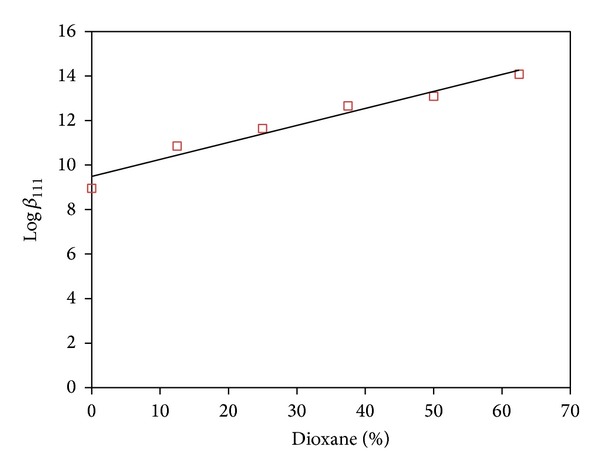
Effect of dioxane on the CBDCA complex formation constant, Log*β*_111_.

The hydrolysis of [Pd(MME)(H_2_O)_2_]^2+^ complex in dioxane-water solution leads to the formation of mono- and dihydroxy species. The dihydroxo bridged dimer was not detected. The p*K*_a_ values of CBDCA and those of the coordinated water molecules in [Pd(MME)(H_2_O)_2_]^2+^ increase linearly with increasing of dioxane concentration. This may be correlated with the ability of a solvent of relatively low dielectric constant to increase the electrostatic attraction between the proton and ligand anion in case of CBDCA and that between a proton and the hydrolyzed form of Pd(II) species. The variation in stability constant of the [Pd(MME)(H_2_O)_2_]^2+^ complex with CBDCA as a function of solvent composition of range from 12.5 to 62.5% is investigated. The results are displayed in Figures 3 and 4. The stability constant for the Pd(MME)-CBDCA complex increases with increasing dioxane concentration. This is explained in terms of complex formation involving oppositely charged ions as in the Pd(MME)-CBDCA complex, which is favored by the low dielectric constant of the medium, that is, with increasing dioxane concentration. The results show that the CBDCA complex with [Pd(MME)(H_2_O)_2_]^2+^ will be more favored in biological environments of lower dielectric constant. It is emphasized that the trend of stability constants as a function of solvent dielectric constant is particularly evident when charged reactants are involved in complex formation.

## 4. Conclusion

Pd(MMA)Cl_2_ complex was synthesised and characterized. In comparison of stability constants of [Pd(MME)(H_2_O)_2_]^2+^ complexes with biorelevant ligands, it would be possible to evaluate the speciation of Pd(II) complexes in biological fluid. This would form a clear basis for understanding the mode of action of such metal species under physiological condition.

Pd(MME)(CBDCA) is taken as a model for carboplatin drug. The study of its stability in dioxane-water solutions of different compositions could be of biological significance. The results show that the formation of the complex is more favoured in biological environments of lower dielectric constant.

## Figures and Tables

**Table 1:** Formation constants for complexes of [Pd(MME)(H_2_O)_2_]^2+^ with amino acids at 25°C and 0.1 M ionic strength.

System	M	L	H^a^	log⁡*β* ^b^	p*K*_a_^c^
Pd(MME)-OH	1	0	−1	−5.29 (0.01)	5.29
	1	0	−2	−12.96 (0.05)	7.67
	2	0	−1	−2.46 (0.07)	

Glycine	0	1	1	9.60 (0.01)	9.60
	0	1	2	11.93 (0.02)	2.33
	1	1	0	10.40 (0.03)	

Alanine	0	1	1	9.69 (0.01)	9.69
	0	1	2	11.89 (0.02)	2.20
	1	1	0	10.33 (0.05)	

Proline	0	1	1	10.52 (0.01)	10.52
	0	1	2	12.03 (0.04)	1.51
	1	1	0	10.79 (0.02)	

*β*-Phenyl alanine	0	1	1	9.12 (0.01)	9.12
	0	1	2	11.01 (0.02)	1.89
	1	1	0	9.87 (0.04)	

Imidazole	0	1	1	7.04 (0.01)	7.04
	1	1	0	8.41 (0.04)	
	1	2	0	15.93 (0.06)	

Lysine	0	1	1	10.51 (0.00)	10.51
	0	1	2	19.71 (0.01)	9.20
	1	1	0	11.15 (0.01)	10.17
	1	1	1	20.29 (0.07)	9.14

Methionine	0	1	1	8.76 (0.00)	8.76
	1	1	0	9.05 (0.04)	

Histamine	0	1	1	9.88 (0.01)	9.88
	0	1	2	15.30 (0.01)	5.42
	1	1	0	12.54 (0.04)	3.43
	1	1	1	15.97 (0.1)	3.43

Histidine	0	1	1	9.52 (0.01)	9.52
	0	1	2	15.81 (0.02)	6.29
	1	1	0	14.26 (0.07)	
	1	1	1	18.30 (0.09)	4.0

^a^M, L, and H are the stoichiometric coefficients corresponding to Pd(MME), amino acid, and H^+^, respectively; the coefficient −1 refers to a proton loss; ^b^log⁡*β* of Pd(MME)-amino acids complexes, standard deviations are given in parentheses; sum of square of residuals is less than 2*E* − 7; ^c^the p*K*_a_ of the ligands and the protonated complexes.

**Table 2:** Formation constants for complexes of [Pd(MME)(H_2_O)_2_]^2+^ with peptides at 25°C and 0.1 M ionic strength.

System	M	L	H^a^	log⁡*β* ^b^	p*K*_a_^c^
Glycinamide	0	1	1	7.88 (0.02)	7.88
	1	1	0	7.25 (0.05)	
	1	1	−1	3.96 (0.01)	3.29

Glycylglycine	0	1	1	7.94 (0.01)	7.94
	0	1	2	10.80 (0.01)	2.86
	1	1	0	8.55 (0.04)	
	1	1	−1	3.17 (0.06)	5.38

Asparagine	0	1	1	8.56 (0.01)	8.56
	1	1	0	9.11 (0.03)	
	1	1	−1	1.63 (0.04)	7.48

Glutamine	0	1	1	9.50 (0.01)	9.50
	1	1	0	9.78 (0.06)	
	1	1	−1	0.93 (0.01)	8.85

^a^M, L, and H are the stoichiometric coefficients corresponding to Pd(MME), peptides, and H^+^, respectively; the coefficient −1 refers to a proton loss; ^b^log⁡*β* of Pd(MME)-peptide complexes, standard deviations are given in parentheses and sum of square of residuals is less than 5*E* − 7; ^c^the p*K*_a_ of the peptides or of coordinated peptides.

**Table 3:** Formation constants for complexes of [Pd(MME)(H_2_O)_2_]^2+^ with dibasic acids at 25°C and 0.1 M ionic strength.

System	M	L	H^a^	log⁡*β* ^b^	p*K*_a_^c^
Cyclobutane-1,1-dicarboxylic acid	0	1	1	5.42 (0.00)	5.42
	0	1	2	8.06 (0.01)	2.64
	1	1	0	7.13 (0.01)	
	1	1	1	8.95 (0.07)	1.82

Malonic acid	0	1	1	5.30 (0.00)	5.30
	0	1	2	7.86 (0.01)	2.56
	1	1	0	6.84 (0.06)	
	1	1	1	10.29 (0.08)	3.45

Oxalic acid	0	1	1	3.83 (0.00)	3.83
	0	1	2	5.66 (0.01)	1.83
	1	1	0	7.16 (0.04)	
	1	1	1	10.3 (0.05)	3.14

Succinic acid	0	1	1	5.21 (0.00)	5.21
	0	1	2	9.26 (0.00)	4.05
	1	1	0	4.27 (0.03)	
	1	1	1	8.45 (0.08)	4.18

Adipic acid	0	1	1	5.11 (0.03)	5.11
	0	1	2	9.35 (0.04)	5.24
	1	1	0	4.11 (0.02)	
	1	1	1	0.494 (0.04)	3.62

^a^M, L, and H are the stoichiometric coefficients corresponding to Pd(MME), dibasic acids, and H^+^, respectively. ^b^log⁡*β* of Pd(MME)-dibasic acid complexes; standard deviations are given in parentheses; sum of square of residuals is less than 1.57*e* ^−8^. ^c^The p*K*_a_ of ligands or the protonated complexes.

**Table 4:** Effect of solvent (dioxane) on the stability constant of Pd(MME)-CBDCA complexes.

System	% Solvent (v/v)	M	L	H	log⁡*β*
Pd(MME)-CBDCA	12.5	0	1	1	6.24 (0.01)
		0	1	2	9.76 (0.02)
		1	0	−1	−4.07 (0.01)
		1	0	−2	−9.63 (0.03)
		2	0	−1	−1.08 (0.04)
		1	1	0	8.19 (0.03)
		1	1	1	10.84 (0.07)
	25.0	0	1	1	6.86 (0.03)
		0	1	2	10.72 (0.05)
		1	0	−1	−3.67 (0.09)
		1	0	−2	−8.93 (0.02)
		2	0	−1	−0.59 (0.09)
		1	1	0	8.69 (0.03)
		1	1	1	11.64 (0.07)
	37.5	0	1	1	7.16 (0.01)
		0	1	2	11.33 (0.02)
		1	0	−1	−3.86 (0.01)
		1	0	−2	−9.13 (0.01)
		2	0	−1	−0.78 (0.40)
		1	1	0	9.07 (0.04)
		1	1	1	12.65 (0.06)
	50.0	0	1	1	7.42 (0.01)
		0	1	2	11.85 (0.01)
		1	0	−1	−3.87 (0.01)
		1	0	−2	−9.40 (0.02)
		2	0	−1	−0.43 (0.07)
		1	1	0	9.08 (0.04)
		1	1	1	13.09 (0.05)
	62.5	0	1	1	7.88 (0.01)
		0	1	2	12.78 (0.02)
		1	0	−1	−3.85 (0.02)
		1	0	−2	−9.80 (0.02)
		2	0	−1	−0.30 (0.04)
		1	1	0	9.8 (0.01)
		1	1	1	14.08 (0.02)
